# Association of GRACE Risk Score with Coronary Artery Disease Complexity in Patients with Acute Coronary Syndrome

**DOI:** 10.3390/jcm10102210

**Published:** 2021-05-20

**Authors:** Georgios Sofidis, Nikolaos Otountzidis, Nikolaos Stalikas, Efstratios Karagiannidis, Andreas S. Papazoglou, Dimitrios V. Moysidis, Eleftherios Panteris, Olga Deda, Anastasios Kartas, Thomas Zegkos, Paraskevi Daskalaki, Niki Theodoridou, Leandros Stefanopoulos, Haralambos Karvounis, Helen Gika, Georgios Theodoridis, Georgios Sianos

**Affiliations:** 1 First Department of Cardiology, AHEPA University Hospital, Aristotle University of Thessaloniki, St. Kiriakidi 1, 54636 Thessaloniki, Greece; g_sofidis@yahoo.gr (G.S.); nickotountzidis@gmail.com (N.O.); nstalik@gmail.com (N.S.); stratoskarag@gmail.com (E.K.); anpapazoglou@yahoo.com (A.S.P.); dimoysidis@gmail.com (D.V.M.); tkartas@gmail.com (A.K.); zegkosth@gmail.com (T.Z.); paraskevi_daskalaki@hotmail.com (P.D.); nikitheodor2@gmail.com (N.T.); hkarvounis@gmail.com (H.K.); 2 Laboratory of Forensic Medicine and Toxicology, School of Medicine, Aristotle University of Thessaloniki, 54124 Thessaloniki, Greece; eleftherios.panteris@gmail.com (E.P.); oliadmy@gmail.com (O.D.); gkikae@auth.gr (H.G.); 3 Biomic_AUTh, Center for Interdisciplinary Research and Innovation (CIRI-AUTH), Balkan Center, B1.4, 10th km Thessaloniki-Thermi Rd, P.O. Box 8318, 57001 Thessaloniki, Greece; gtheodor@chem.auth.gr; 4 Lab of Computing, Medical Informatics and Biomedical Imaging Technologies, Aristotle University of Thessaloniki, 54124 Thessaloniki, Greece; lstefano@auth.gr; 5 Laboratory of Analytical Chemistry, Department of Chemistry, Aristotle University of Thessaloniki, 54124 Thessaloniki, Greece

**Keywords:** acute coronary syndrome, GRACE score, coronary angiography, SYNTAX score

## Abstract

The GRACE score constitutes a useful tool for risk stratification in patients with acute coronary syndrome (ACS), while the SYNTAX score determines the complexity of coronary artery disease (CAD). This study sought to correlate these scores and assess the accuracy of the GRACE score in predicting the extent of CAD. A total of 539 patients with ACS undergoing coronary angiography were included in this analysis. The patients were classified into those with a SYNTAX score < 33 and a SYNTAX score ≥ 33. Spearman’s correlation and receiver operator characteristic analysis were conducted to investigate the role of the GRACE score as a predictor of the SYNTAX score. There was a significantly positive correlation between the SYNTAX and the GRACE scores (r = 0.32, *p* < 0.001). The GRACE score predicted severe CAD (SYNTAX ≥ 33) moderately well (the area under the curve was 0.595 (0.522–0.667)). A GRACE score of 126 was documented as the optimal cut-off for the prediction of a SYNTAX score ≥ 33 (sensitivity = 53.5% and specificity = 66%). Therefore, our study reports a significantly positive correlation between the GRACE and the SYNTAX score in patients with ACS. Notably, NSTEMI patients with a high-risk coronary anatomy have higher calculated GRACE scores. A multidisciplinary approach by a heart team could possibly alter the therapeutic approach and management in patients presenting with ACS and a high calculated GRACE score.

## 1. Introduction

Acute coronary syndrome (ACS) constitutes the leading cause of morbidity and mortality worldwide [1,2]. Prospective risk stratification in patients with ACS, including unstable angina (UA), non-ST-segment elevation myocardial infarction (NSTEMI), and ST-segment elevation myocardial infarction (STEMI), facilitates decisions on the timing of angiography and thus enables a personalized therapeutic strategy [3,4]. Current American and European clinical guidelines suggest the use of a Global Registry of Acute Coronary Events (GRACE) risk score as a predictor of major adverse events in these patients [5,6,7]. This risk stratification model, which consists of various clinical, laboratory, and electrocardiographic parameters that are documented on admission, is commonly used in clinical practice to estimate the risk of death or myocardial infarction within six months, including in-hospital events. Moreover, the most recent guidelines on non-ST-elevation ACS patients recommend the GRACE score as a tool for the identification of high-risk patients who will benefit from an early invasive strategy [8]. Nevertheless, it is not intended to identify the complexity of CAD.

The Synergy Between Percutaneous Coronary Intervention (SYNTAX) score, a comprehensive angiographic grading tool that takes into account anatomic risk factors, is the best-known scoring system to assess the extent of CAD [9]. Based on the complexity of CAD, this score is capable of objectively guiding decision-making between coronary artery bypass grafting (CABG) surgery and percutaneous coronary intervention (PCI) [10]. Nonetheless, the SYNTAX score relies on invasive coronary angiography (CA) findings to calculate the coronary anatomic complexity. Consequently, non-invasively estimating the complexity of CAD prior to CA could change the prognostic plan, the timing, and the intensity of intervention, and possibly integrate a multidisciplinary approach by both interventional cardiologists and cardiac surgeons in patients with severe CAD.

To date, several studies have investigated the correlation between risk-stratification scoring systems and the severity of CAD [11,12,13,14,15,16]. To our knowledge, only a limited number of them used the GRACE score to appreciate its reliability in predicting high-risk CAD and most of them solely enrolled patients with non-ST-elevation ACS [12,17,18,19]. However, the GRACE score could also highlight the need for an early transfer of STEMI patients from non-capable PCI hospitals to a PCI-center just after thrombolytic treatment [20]. The aim of this study was to thoroughly investigate the association between the GRACE and the SYNTAX scores in patients presenting with ACS (STEMI, NSTEMI, and UA). Thereby, we aspire to explore the angiographic severity of CAD encountered across the GRACE score continuum and ultimately recommend a GRACE score value that is predictive of complex CAD, which will be the cut-off to consider a more aggressive multidisciplinary therapeutic approach.

## 2. Materials and Methods

### 2.1. Study Design

This single-center cross-sectional study originated from the CorLipid trial (Correlation of Clinical Types and Complexity of Coronary Artery Disease with Patients’ Metabolic Profile, ClinicalTrials.gov Identifier: NCT04580173). Briefly, the CorLipid trial aimed to investigate the association of patients’ metabolic fingerprints with the severity of CAD in patients undergoing CA. The trial’s design and detailed inclusion and exclusion criteria have been reported previously [21].

### 2.2. Study Population

The present study enrolled 539 adult patients presenting to the AHEPA University Hospital in Thessaloniki with ACS and were undergoing CA between July 2019 and December 2020. ACS was diagnosed according to the fourth universal definition of Acute Myocardial Infarction [22]. Patients that had previously had a coronary revascularization procedure were excluded from the study. Every study participant provided written informed consent prior to enrollment. The protocol of this study was approved by the Ethics and Scientific Committee of AHEPA University Hospital (reference number 12/13-06-2019) and conforms to the Declaration of Helsinki [23].

For each patient, demographics and baseline clinical characteristics, as well as the determinants of the GRACE risk score 2.0 [24] (i.e., age, heart rate, systolic blood pressure, serum creatinine concentration, the presence of ST-segment deviation, cardiac arrest during admission, elevated serum cardiac biomarkers, and Killip class) were documented on admission and assessed by two experienced cardiologists based on the patients’ medical records. The GRACE 2.0 ACS risk calculator is available online (https://qxmd.com/calculate/calculator_262/grace (accessed on 9 May 2021)) and implements the revised GRACE algorithms for predicting death or death/myocardial infarction following initial ACS.

### 2.3. Coronary Angiography and Scores Calculation

Patients underwent CA in our catheterization laboratory and all CAs were visually assessed by two well-experienced interventional cardiologists (GS1, GS2), blinded to all other clinical data. The percentage of diameter stenosis and lesion length were calculated using quantitative coronary angiography (Quantcor QCA Software, Siemens Medical Solutions). The SYNTAX score algorithm was used to assess the complexity of coronary anatomy in the patients with STEMI, NSTEMI, or UA, according to standardized practices [9,25]. A SYNTAX score cut-off level of 33 was used to distinguish the patients presenting with severe lesion complexity (high-risk coronary anatomy) because CABG offers significantly better outcomes than PCI at one and five years in these patients. [9,10,26].

### 2.4. Statistical Analysis

Continuous data are presented as mean ± standard deviation and were compared via the Student’s *t*-test or Mann–Whitney test. Categorical variables are expressed as frequency and percentages and were compared via the chi-square test. The Kruskal–Wallis H test was used to compare two or more groups of an independent variable on a continuous or ordinal dependent variable. The inter- and intra-observer variability of the GRACE and SYNTAX scores were assessed based on the data obtained from a subset of 54 subjects (10% of study population), performing Spearman’s correlations, and the intraclass correlation coefficient [27,28].

Spearman’s correlation analysis was further executed to investigate the correlation between the GRACE score and the SYNTAX score. The correlation coefficient was considered weak if <0.5, moderate if between 0.5 and 0.7, and strong if >0.7. Receiver operating characteristic (ROC) analysis was performed to determine the predictive accuracy of the GRACE risk score regarding a high-risk coronary anatomy (SYNTAX score ≥ 33). A prediction would be regarded as significant if the area under the ROC curve (AUC) was statistically different from 0.5. Furthermore, the sensitivity and specificity of the GRACE score as a predictor of severe CAD were calculated. A GRACE score >140 is frequently defined as a high-risk indicator for adverse clinical outcomes, whereas a score of 1–108 or 109–140 are defined as low or intermediate predictors of clinical outcomes, respectively.

Linear regression analysis using stratified bootstrapping to account for the non-parametric nature of the data was performed to identify independent predictors of a high SYNTAX score. R, R^2^, Durbin–Watson, and Nagelkerke R^2^ metrics, along with *p*-values, are reported for the linear and logistic models, respectively. The factors significantly associated with severe CAD (SYNTAX score ≥ 33) in univariate analysis were further involved in the multivariate model to identify additional clinical predictors of severe CAD, apart from those included in the GRACE score. The level of statistical significance was set to 0.05 and the statistical analysis was performed using SPSS (IBM SPSS Statistics for Windows, Version 26.0. IBM Corp: Armonk, NY, USA) software.

## 3. Results

### 3.1. Population Characteristics

A total of 539 patients (76% men) with confirmed ACS (STEMI, NSTEMI, and UA) who underwent a primary PCI were enrolled in the present analysis. The mean age of participants was 63 ± 13 years, and 53.6% were smokers (Table 1). Of these patients, 31.9% suffered from dyslipidemia and almost one in five from diabetes mellitus (DM). In addition, 223 (41.4%) patients were diagnosed with STEMI, 173 (32.1%) with NSTEMI, and 141 (26.2%) with UA. According to the calculated GRACE score on admission, 227 (42.1%) patients were considered as low-risk (GRACE score < 108), 168 (31.2%) were identified as intermediate-risk (GRACE score = 109–140), and 144 (26.7%) as high-risk (GRACE score > 140). Finally, the mean GRACE score was equal to 116 ± 38.

A visual assessment of the CAs showed that most patients (65.9%) had 1–3 atherosclerotic lesions. At least one totally occluded artery was present in 34.1% of patients, whereas 16.1% patients had no critical coronary arterial narrowing. Three-vessel disease was identified in 25.2% of the sample, two-vessel disease in 32.5% of the sample, and single-vessel disease in 26.2%. Moreover, heavy calcification and thrombus were present in 28.8% and 31.7% of the patients, respectively. The mean SYNTAX score was 16.2 ± 13.4. The majority of the population (72.7%) had a low SYNTAX score (<22), whereas 15.8% of patients had a score of 22–32, and only 11.5% of patients had a SYNTAX score ≥33. When comparing patients with a SYNTAX score ≥33 to patients with a SYNTAX score <33, DM and a history of hypertension were more frequent among the patients with a SYNTAX score ≥33 (*p* = 0.013 and *p* < 0.001, respectively). Increased GRACE scores, urea levels, and age were identified in patients with a SYNTAX score ≥33, whereas the glomerular filtration rate and left ventricular ejection fractions were significantly less in this patient group.

With regard to the reproducibility of the score calculations, intra-observer and interobserver reliabilities were high for both the GRACE (interobserver: 0.88; intra-observer: 1.00) and SYNTAX scores (interobserver: 0.82; intra-observer: 0.95). Interclass correlation coefficients for the GRACE and SYNTAX scores were equal to 0.89 (95% C.I.: 0.88–0.99) and 0.83 (95% C.I.: 0.81–0.98), respectively.

### 3.2. Correlation between the GRACE Score and Coronary Artery Disease Complexity

**Figure 1:**
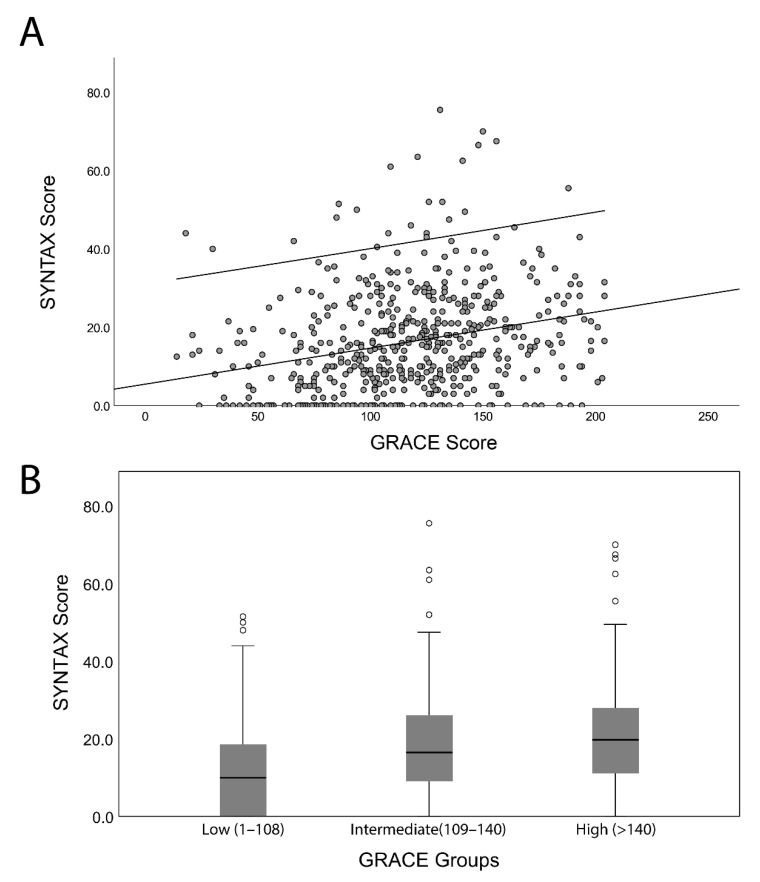
(**A**) Correlation of the GRACE score with the SYNTAX score. (**B**) Boxplot of the SYNTAX score for the three GRACE score (low-, intermediate-, and high-risk) groups.

Spearman’s correlation analysis demonstrated that there was a significant but weak positive correlation between the GRACE and the SYNTAX scores (r = 0.32, *p* < 0.001) (Figure 1A). Additionally, the median SYNTAX score of high-risk patients (GRACE score > 140) was greater than that of patients with intermediate (GRACE score = 109–140) and low (GRACE score < 109) GRACE scores on admission (*p* < 0.001), as illustrated in the boxplots in Figure 1B. Nevertheless, there was no statistical difference between the low- and intermediate-risk groups (*p* = 0.77).

**Figure 2:**
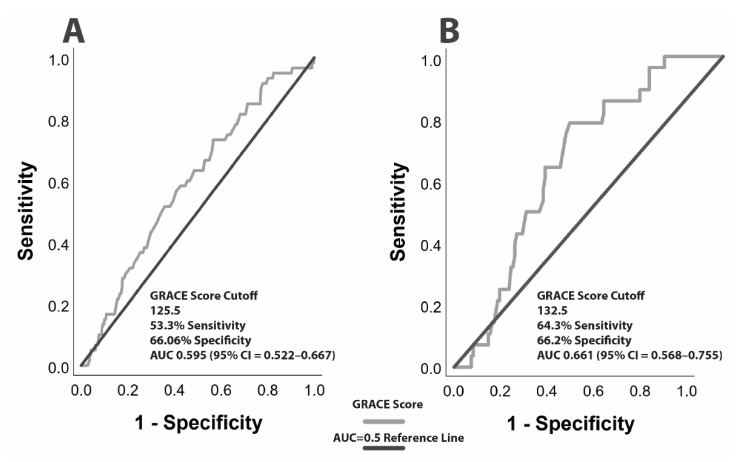
ROC curve on the predictive significance of the GRACE score for high-complexity CAD (SYNTAX score ≥ 33): (**A**) in ACS patients (AUC = 0.595, 95% CI = 0.522–0.667) and (**B**) in NSTEMI patients (AUC = 0.661, 95% CI = 0.568–0.755).

The ROC curves demonstrate that the GRACE score has a significant discriminative ability to predict high CAD complexity (SYNTAX ≥ 33) both in patients with ACS (2A) and in those with NSTEMI (2B), with an AUC of 0.595 and a 95% CI = 0.522–0.667, and an AUC of 0.661 and a 95% CI = 0.568–0.755, respectively (Figure 2). A GRACE score equal to 126 was documented as the optimal cut-off for the prediction of a SYNTAX score ≥33 in patients with ACS (sensitivity = 53.5% and specificity = 66%), whereas a GRACE score of 133 is regarded as the optimal cut-off value for predicting high complexity CAD in NSTEMI patients (sensitivity = 64% and specificity = 66%).

On the other hand, the GRACE score could not significantly predict severe CAD in patients with STEMI (AUC = 0.510, 95% CI = 0.361–0.659) and in those with UA (AUC = 0.585, 95% CI = 0.435–0.735).

### 3.3. Additional Clinical Predictors of Severe Coronary Artery Disease

**Figure 3:**
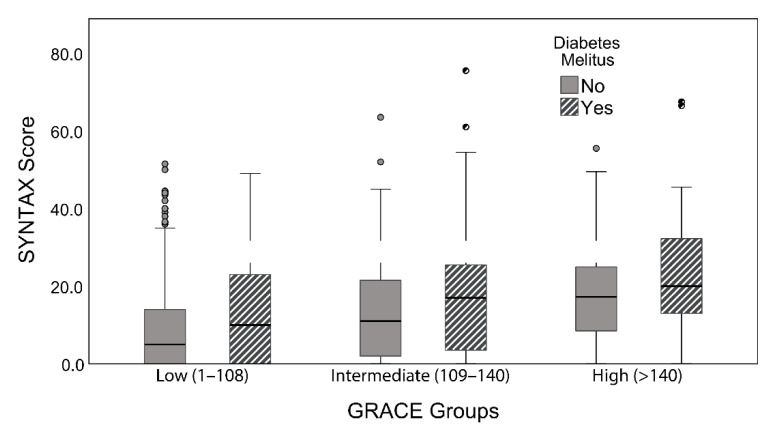
The median SYNTAX score of diabetic patients was higher than that of non-diabetic patients among the three different classes (low, intermediate, and high) of the calculated GRACE score.

Bootstrapped multivariate analysis demonstrated that DM (*p* = 0.002), male gender (*p* = 0.020), and GRACE risk scores (*p* = 0.001) independently predicted the presence of severe CAD (SYNTAX score ≥ 33) (Table 2). In addition, across the three different classes (low, intermediate, and high) of the calculated GRACE scores, the median SYNTAX score of diabetic patients was higher than that of non-diabetic patients (Figure 3). Interestingly, patients with DM were found to have an increased GRACE score during ACS presentation (DM: 125.6 ± 35.1 vs. non-DM: 113 ± 39.5 GRACE score, *p* = 0.001).

## 4. Discussion

The main finding of this study is the presence of a weak yet significant positive correlation between the GRACE and the SYNTAX scores in ACS patients, especially in those with NSTEMI. A higher GRACE score (>126 in ACS patients and >133 in NSTEMI patients) was found to be predictive of a higher coronary anatomical complexity (SYNTAX score ≥ 33). Moreover, DM was identified as an independent predictor of a higher CAD complexity, which is not included as a determinant of the GRACE score.

The utility of the GRACE and the SYNTAX scores in clinical practice has been previously described [3,24,29,30,31]. Both scores have been found to independently predict cardiovascular death in patients with ACS [32,33,34,35]. Moreover, a combination of the two algorithms (SYNTAX–GRACE score) has also been described and revealed to be of higher prognostic value for in-hospital cardiovascular death compared to the SYNTAX score alone [33].

Several studies have previously reported the positive correlation between the GRACE score and other CAD severity assessment tools [12,13,16,17,18,19,36,37,38,39,40]. Specifically, Hammami et al. found that the GRACE score was significantly higher in UA and NSTEMI patients with a SYNTAX score ≥ 33, with a weak positive correlation reported between the two scores (r = 0.23) [16]. Nevertheless, the ROC analysis did not confirm the predictive value of the GRACE score for a SYNTAX score ≥ 33. In another study, Rahmani et al. found a positive but also weak correlation between the SYNTAX and the GRACE scores (r = 0.34, *p* < 0.001) in UA and NSTEMI patients [19]. A study on a similar patient population conducted by Avci et al. revealed a significant positive correlation between the two scores (r = 0.338, *p* < 0.001), while a GRACE score of 123 was the optimal cut-off point in order to identify patients with a SYNTAX score ≥33 [18].

Hitherto, to our knowledge, evidence on the correlation between the GRACE and the SYNTAX scores in patients with ACS, including STEMI, is scarce, with only one study demonstrating a weak positive correlation between the two scores (r = 0.423, *p* < 0.001) [13]. Our study provides additional information to corroborate the existing correlation between the two scores (r = 0.32, *p* < 0.001). The ROC analysis showed that the diagnostic value of the GRACE score for severe CAD was significant in the total ACS population and especially in the subgroup of NSTEMI patients. The most recent clinical guidelines suggest that in non-ST-elevation ACS patients with a high GRACE score, an early (<24 h) invasive strategy is beneficial [8]. Therefore, according to the present study, NSTEMI patients applicable for an early invasive approach present a more complex CAD, indicating the necessity for a multidisciplinary heart team approach.

These findings might not be applicable in the case of STEMI patients. This could be explained by the fact that the SYNTAX score is not always increased in patients with STEMI, and the calculation of the SYNTAX score was performed using a modified strategy for scoring the infarct-related artery (IRA) [25]. In addition, the existence of further parameters, apart from CAD complexity (e.g., pain-to-balloon time and thrombus burden), could affect outcomes in STEMI patients [41,42]. However, a high GRACE score in STEMI patients presenting to non-PCI capable hospitals could be an important indicator for an early transfer to a PCI-center just after thrombolytic treatment.

Furthermore, DM constitutes an independent prognostic factor in complex coronary anatomy that is not included in the GRACE score [43,44,45]. Notably, a significantly higher complexity of CAD was observed in diabetic patients, in comparison with non-diabetic subjects in the same risk group, according to the GRACE score. This finding is also highlighted in a study conducted by Cakar et al., where the Gensini, instead of the SYNTAX, score was used as an angiographic tool grading the complexity of CAD [12]. Therefore, we speculate that DM could be embedded in the GRACE score to increase its discriminative power for a better risk assessment of ACS patients in future studies.

Our findings suggest that the use of the GRACE score in clinical practice could not only assess the occurrence of in-hospital and 6-month to 3-year mortality in ACS patients, but could also provide clues on the anticipated severity of CAD, prior to undergoing CA. Hence, during a clinical assessment of ACS (especially NSTEMI) patients in the emergency department, a higher GRACE score could indicate severe CAD, and thus alert the clinicians to provide a more aggressive therapeutic approach or an early transfer to a PCI-capable center.

This study should be interpreted in the context of certain limitations. We should not discount the single-center character of the study, and the small number of patients with a high CAD complexity. The heterogeneity of the UA subpopulation, as well as specific modifiers encountered in STEMI patients (e.g., the modified method of SYNTAX score calculation for rating the IRA) might have contributed to generating non-significant findings in these patients. We should also acknowledge that the SYNTAX score was calculated for each patient by the cardiologists who performed the CA, which could potentially lead to a detection bias. Finally, future studies of a larger sample size are warranted to validate and bolster the significance of our findings.

## 5. Conclusions

Our study reports a significantly positive correlation between the GRACE and the SYNTAX scores in patients with ACS undergoing CA. Notably, NSTEMI patients with a high-risk coronary anatomy have a higher calculated GRACE score. However, the ability of the GRACE score to predict high-risk coronary anatomy is modest. A multidisciplinary approach by a heart team could possibly alter the therapeutic approach and management in patients presenting with ACS, especially those with NSTEMI and a high GRACE score. Prospective large-scale studies are warranted to validate our findings.

## Figures and Tables

**Table 1:** Demographics and baseline clinical characteristics of study participants.

Parameters	SYNTAX < 33 (*Ν* = 477)	SYNTAX ≥ 33 (*Ν* = 62)	*p*-Value
***Demographics***			
**Female Sex–No (%)**	115 (24.2%)	14 (23.3%)	0.87
**Age (Years)–mean (±SD)**	62 (±13.0)	69 (±11.0)	**<0.01**
**Body Mass Index (Kg/m^2^)–mean (±SD)**	28.3 (±4.5)	28.6 (±4.7)	0.63
***Coronary Heart Disease risk factors***			
**Diabetes Mellitus–No (%)**	106 (22.3%)	25 (41.7%)	**<0.01**
**Hypertension–No (%)**	244 (51.4%)	41 (68.3%)	**0.01**
**Dyslipidemia–No (%)**	150 (31.6%)	20 (33.3%)	0.79
**Smoking–No (%)**	257 (54.1%)	29 (48.3%)	0.39
**Family history–No (%)**	99 (20.8%)	11 (18.3%)	0.64
***Medical History/Underlying Diseases***			
**Chronic kidney Disease–No (%)**	24 (5.1%)	6 (10.0%)	0.11
**Atrial Fibrillation–No (%)**	38 (8.0%)	5 (8.3%)	0.92
**Previous stroke–No (%)**	13 (2.7%)	1 (1.7%)	0.63
**Aortic Aneurysms–No (%)**	3 (0.6%)	1 (1.7%)	0.38
**Peripheral Vascular Disease–No (%)**	22 (4.6%)	3 (5.0%)	0.89
**Chronic Obstructive Pulmonary Disease–No (%)**	26 (5.5%)	2 (3.3%)	0.48
**Autoimmune Disease–No (%)**	7 (1.5%)	3 (5.0%)	**0.04**
***Risk Scores***			
**GRACE Score–mean (±SD)**	115 (±39)	126 (±37)	**0.01**
**SYNTAX score–mean (±SD)**	12.8 (±9.4)	42.9 (±9.6)	
***Parameters on Admission-Means(±SD)***			
**LVEF (%)–mean (±SD)**	48 (±11)	44 (±11)	**0.01**
**eGFR (mL/min)–mean (±SD)**	95 (±37)	83 (±37)	**0.04**
**Creatinine (mg/dL)–mean (±SD)**	1.09 (±0.93)	1.09 (±1.29)	0.37
**TnThs (ng/dL)–mean (±SD)**	1203 (±209)	1153 (±191)	0.70
**CPK (U/L)–mean (±SD)**	639 (±194)	436 (±69)	0.49
**LDH (U/L)–mean (±SD)**	423 (±46)	393 (±28)	0.84
**Total Cholesterol (mg/dL)–mean (±SD)**	166 (±43)	154 (±46)	0.07
**LDL (mg/dL)–mean (±SD)**	96 (±37)	88 (±42)	0.09
**HDL (mg/dL)–mean (±SD)**	40 (±11)	43 (±14)	0.17

LVEF = left ventricular ejection fraction; eGFR = estimated glomerular filtration rate; TnThs = troponin T high sensitivity; CPK = creatine phosphokinase; LDH = lactate dehydrogenase; LDL = low density lipoprotein; HDL = high density lipoprotein.

**Table 2:** Bootstrapped multivariate analysis for the prediction of severe coronary artery disease (SYNTAX score ≥ 33).

Bootstrapped Multivariate Analysis		95% CI		
Variables	Beta	Lower	Upper	*p* -Value (2-Tailed)
**Diabetes Mellitus**	4.738	1.909	7.402	**0.002**
**Age**	0.106	0.014	0.200	**0.027**
**Gender**	2.979	0.409	5.575	**0.020**
**GRACE Groups**	3.261	1.711	4.769	**0.001**

**Model metrics:** R = 0.333, adjusted R^2^ = 0.104, Durbin–Watson: 0.130.

## Data Availability

Data are available from Georgios Sianos (e-mail: gsianos@auth.gr) upon reasonable request and with the permission of AHEPA University Hospital.
